# Expression of the zinc finger gene EVI-1 in ovarian and other cancers.

**DOI:** 10.1038/bjc.1996.583

**Published:** 1996-11

**Authors:** D. J. Brooks, S. Woodward, F. H. Thompson, B. Dos Santos, M. Russell, J. M. Yang, X. Y. Guan, J. Trent, D. S. Alberts, R. Taetle

**Affiliations:** Department of Medicine, University of Arizona and Arizona Cancer Center, Tucson 85724, USA.

## Abstract

**Images:**


British Journal of Cancer (1996) 74, 1518-1525
© 1996 Stockton Press All rights reserved 0007-0920/96 $12.00

### Expression of the zinc finger gene EVI-1 in ovarian and other cancers

DJ Brooks[1], S Woodward[1], FH Thompson[1], B Dos Santos[1], M Russell[1], J-M Yang[1], X-Y Guan[2], J Trent[2], DS Alberts[1] and R Taetle[1]

[1]*Departments of Medicine, Pathology and Pharmacology, University of Arizona and Arizona Cancer Center, Tucson, AZ, 85724 and* [2]*National Center for Human Genome Research, NIH, Bethesda, MD, USA.*

**Summary** The *EVI-1* gene was originally detected as an ectopic viral insertion site and encodes a nuclear zinc finger DNA-binding protein. Previous studies showed restricted EVI-1 RNA or protein expression during ontogeny; in a kidney and an edometrial carcinoma cell line; and in normal murine oocytes and kidney cells. EVI-1 expression was also detected in a subset of acute myeloid leukaemias (AMLs) and myelodysplasia. Because EVI-1 is expressed in the urogenital tract during development, we examined ovarian cancers and normal ovaries for EVI-1 RNA expression using reverse transcription-polymerase chain reaction (RT-PCR) and RNAase protection. Chromosome abnormalities were examined using karyotypes and whole chromosome 3 and 3q26 fluorescence *in situ* hybridisation (FISH). RNA from six primary ovarian tumours, five normal ovaries and 47 tumour cell lines (25 ovarian, seven melanoma, three prostate, seven breast and one each of bladder, endometrial, lung, epidermoid and histiocytic lymphoma) was studied. Five of six primary ovarian tumours, three of five normal ovaries and 22 of 25 ovarian cell lines expressed EVI-1 RNA. A variety of other non-haematological cancers also expressed EVI-1 RNA. Immunostaining of ovarian cancer cell lines revealed nuclear EVI-1 protein. In contrast, normal ovary stained primarily within oocytes and faintly in stroma. Primary ovarian tumours showed nuclear and intense, diffuse cytoplasmic staining. Quantitation of EVI-1 RNA, performed using RNAase protection, showed ovarian carcinoma cells expressed 0 to 40 times the EVI-1 RNA in normal ovary, and 0-6 times the levels in leukaemia cell lines. Southern analyses of ovarian carcinoma cell lines showed no amplification or rearrangements involving EVI-1. In some acute leukaemias, activation of EVI-1 transcription is associated with translocations involving 3q26, the site of the *EVI-1* gene. Ovarian carcinoma karyotypes showed one line with quadruplication 3(q24q27), but no other clonal structural rearrangements involving 3q26. However, whole chromsome 3 and 3q26 FISH performed on lines with high EVI-1 expression showed translocations involving chromosome 3q26. EVI-1 is overexpressed in ovarian cancer compared with normal ovaries, suggesting a role for EVI-1 in solid tumour carcinogenesis or progression. Mechanisms underlying EVI-1 overexpression remain unclear, but may include rearrangements involving chromosome 3q26.

**Keywords:** zinc finger; ovarian cancer; EVI-1

Ovarian cancer is the leading cause of death from female genitourinary cancers in the United States (Boring *et al.*, 1994). Compared with other solid tumours, relatively little is known regarding the molecular pathogenesis and progression of ovarian cancers. Mutations and/or altered expression of p53 (Marks *et al.*, 1991; Milner *et al.*, 1993) and HER-2/*neu* (Rubin *et al.*, 1993, 1994) occur frequently, but *ras* mutations are relatively infrequent (van't Veer *et al.*, 1988; Smith *et al.*, 1989) compared with other solid tumours. Sporadic reports of abnormal expression of other proto-oncogenes, including *myc*, *fos*, *AKT2* and *int-2* have appeared (Sasano *et al.*, 1990; Cheng *et al.*, 1992), but the roles of these genes in ovarian carcinogenesis and the relative frequencies of their expression in ovarian cancers remain unclear.

The *EVI-1* (ecotropic virus integration-1) gene was originally detected as a murine virus insertion site in experimental leukaemias (Mucenski *et al.*, 1988) and localised to human chromosome band 3q26 (Morishita *et al.*, 1990*a*). The gene encodes a nuclear zinc finger protein with specific DNA-binding properties (Matsugi *et al.*, 1990; Perkins *et al.*, 1991*a*; Delwel *et al.*, 1993). The function of *EVI-1* in normal cells remains unknown, but it is sequentially expressed in limb buds during ontogeny, suggesting a role in cell migration (Perkins *et al.*, 1991*b*). During embryological development in mice, EVI-1 protein is expressed at high levels in restricted sites including the urinary and Mullerian systems, bronchial epithelium, focal areas in nasal cavities, endocardial cushions, and developing limbs (Perkins *et al.*, 1991*b*). Recent data suggest EVI-1 alters transcription directed by other Zn finger genes, such as GATA-1 (Kreider *et al.*, 1993) and regulates c-*fos* expression (Tanaka *et al.*, 1994), thus controlling gene expression.

In normal adult murine tissues, EVI-1 expression was previously detected in murine kidney tubules and oocytes (Perkins *et al.*, 1991*b*; Morishita *et al.*, 1990*c*). The presence of EVI-1 protein has also been demonstrated in human kidney and endometrial tissues (Kreider *et al.*, 1993) and EVI-1 RNA was induced in short-term culture of kidney tubule cells by cAMP (Bartholomew and Clark, 1994). Using Northern analysis, expression of EVI-1 RNA was detected in only a few human solid tumour cell lines (Morishita *et al.*, 1990*b*).

Because EVI-1 is expressed in developing urogenital tract and normal oocytes, we examined its expression in normal human ovary and ovarian cancers. Using reverse transcription-polymerase chain reaction (RT-PCR), EVI-1 RNA was detected in the vast majority of ovarian cancers and in many other solid tumours. Some ovarian tumours expressed extremely high levels of EVI-1 RNA compared with normal ovary, suggesting that the *EVI-1* gene may play a role in pathogenesis or progression of ovarian cancers.

### Methods

### *Tumour specimens*

Primary ovarian cancer tissue specimens were obtained from untreated patients (Thompson *et al.*, 1994). All specimens, including those used to establish cell lines from tumours, were obtained as excess pathological material using procedures approved by the Committee on Human Subjects, University of Arizona. Primary tumours and normal ovaries were stored frozen at −70°C until used. Four of five

Correspondence: DJ Brooks, Section of Hematology/Oncology, Arizona Cancer Center, 1515 North Campbell Ave., PO Box 245024, Tucson, AZ 85724-5024, USA
Received 11 March 1996; revised 23 May 1996; accepted 28 May 1996

normal ovaries studied contained both oocytes and stromal elements. The fifth, obtained from a post-menopausal patient, contained no oocytes. Previously established cancer cell lines were maintained using RPMI-1640 medium with either 5% or 10% fetal bovine serum (FBS), except the HEC1b endometrial carcinoma (Morishita *et al.*, 1990*b*) line, which was maintained in minimum essential median (MEM) with 10% FBS. These lines included: OVCAR3 (Cheng *et al.*, 1992), SKOV3 (Buick *et al.*, 1985), 2008 (Naredi *et al.*, 1994), 2780 (Mann *et al.*, 1991), 367 (Taetle and Honeysett, 1987) and Colo-316 (Woods *et al.*, 1979). Ovarian carcinoma cell lines established from primary tumours were designated UACC (numbers 469, 1598, 326, 188, 591, 1763, 2614, 1703, 2996, 2983, 2727, 295, 1499, 2980, 1123, 2982, 341, 2912 and 2661) and characterised and maintained as previously described (Leibowitz, 1986). Melanoma cell lines developed from primary tumours were also assigned UACC numbers (475, 502 and 2946). In addition, three previously described melanoma lines, 355, 242 (Taetle *et al.*, 1987) and 289 (Taetle and Honeysett, 1987) were tested. With the exception of MDA 468 (Taetle *et al.*, 1988), all breast tumour cell lines were developed at the University of Arizona, and included UACC numbers 2642, 2715, 2436, 245, 812 and 893. All UACC cell lines were established from primary tumour tissue and the original diagnosis confirmed by independent pathology review. Factor-dependent UCSD/AML1 and factor-independent HEL leukaemia cells expressing EVI-1 RNA were maintained as described previously (Russell *et al.*, 1993, 1994; Oval *et al.*, 1990). Other lines studied included prostate [PC3 (Rodan *et al.*, 1983), LN CaP (Wilding *et al.*, 1990), DU 146 (Mickey *et al.*, 1980)], epidermoid [A431 (Taetle *et al.*, 1988)], lung (A375), AML (HL60) and histiocytic lymphoma cells, U937 (Russell *et al.*, 1994).

*Immunostaining*

Carcinoma cell lines were harvested using custom ATV (0.5% trypsin, 12% EDTA, sodium bicarbonate) (Irvine Scientific Santa Ana, CA, USA) trypsin. Cytospin preparations were made on aminopropyltriethoxysilane (Sigma, St Louis, MO, USA)-treated slides for immunostaining. Tumour tissue was frozen or fixed in 10% neutral-buffered formalin and processed in paraffin as routine surgical pathology specimens. Blocks were stored at room temperature.

Formalin-fixed tissue was sectioned on poly-L-lysine-coated slides and baked at 60°C for 20 min immediately before staining. The slides were deparaffinised in xylene for 2 min twice, then rehydrated in 100%, 85% and 70% ethanol. Cytospin preparations were prepared as above, air-dried at 4°C for 1–2 h and fixed in a solution of 1% formalin and 1% Triton X in water for 20 min at room temperature. Fixed slides were washed in two changes of phosphate-buffered saline (PBS) for 3 min each. The slides were then covered with a 1:250 dilution of anti EVI-1 antiserum (Matsugi *et al.*, 1990) (kindly provided by Dr James Ihle, St Jude's Hospital, Memphis, TN, USA) or rabbit serum (1:250; Cappel, Durham, NC, USA) as a negative control, in a dark humidified chamber.

After incubation, slides were rinsed in three changes of 1×PBS for 3 min each. Biotinylated swine anti-rabbit antibody (Dako, Carpinteria, CA, USA) diluted 1:300 was placed on the slides in a dark, humidified chamber at room temperature for 30 min. The slides were then rinsed as described above. EVI-1 protein was detected using alkaline phosphatase or horseradish peroxidase-conjugated streptavidin (Guesdon *et al.*, 1995). Known EVI-1-positive (UCSD/AML1, HEL or HEC1b) and -negative (ovarian 2780 or HL60 leukaemia) cells were stained concurrently.

*RNA polymerase chain reaction*

EVI-1 RNA was detected using reverse transcription–polymerase chain reaction (RT–PCR) as previously described (Russell *et al.*, 1994) with modifications. RNA was prepared by lysis and extraction from cells with Trizol (Gibco BRL, Gaithersberg, MD, USA), according to the manufacturer's instructions. Reverse transcription of approximately 0.1–0.5 $\mu$g RNA was performed for 45 min at 37°C in a solution containing the following components in final concentrations: 1×*Taq* reaction buffer (Boehringer Mannheim) with an additional 5 mM magnesium chloride, 1 mM dNTPs (Perkin Elmer); 2.5 pM $\mu l^{-1}$ Oligo dt15 primer (Boehringer Mannheim); 1 U $\mu l^{-1}$ RNAasin (RNAase inhibitor from Promega); 2.5 U $\mu l^{-1}$ MMLV reverse transcriptase (Gibco/BRL). The amplification mixture contained (final concentrations): 10 mM Tris-HCl at pH 8.3; 1.5 mM magnesium chloride; 50 mM potassium chloride; 3 ng $\mu l^{-1}$ each of 5′ and 3′ primer (Biosynthesis, Lewisville, TX, USA); and *Taq* DNA polymerase at 1.25 units per reaction (Boehringer Mannheim, Indianapolis, IN, USA). The PCR was run for one cycle at 92°C for 5 min, then 30 cycles at 92°C for 1 min, at 62°C for 1 min and at 72°C for 2 min, plus an additional cycle with 15 min extension at 72°C. In each study, UCSD/AML1 or HEL RNA (EVI-1-positive) and HL60 leukaemia cell or 2780 ovarian cancer RNA (EVI-1-negative) plus a mock reaction containing no RNA was performed. Primers spanned bases 223–702 (5′ EVI-1 product) and 2110–2390 (3′ EVI-1 products) of the EVI-1 coding sequence (Morishita *et al.*, 1990*b*). The 3′ EVI-1 reverse PCR yields two characteristic products of 254 and 272 bases owing to alternative splicing (Russell *et al.*, 1994). Histone or GAPDH (available for later experiments) RNA was assessed in parallel to assure RNA integrity (Russell *et al.*, 1994).

Identity of EVI-1 PCR products was verified by probing Southern blots of RT–PCR products with an oligonucleotide internal to the priming sequences (Russell *et al.*, 1993). Both 3′ EVI-1 PCR products from two cell lines, Colo316 and SKOV-3, were also sequenced using automated techniques (Scripps Clinic and Research Foundation, La Jolla, CA, USA), and yielded the expected EVI-1 sequences (Morishita *et al.*, 1990*b*).

*Assessment of EVI-1 RNA expression*

EVI-1 RNA content was evaluated using RNAase protection. For RNAase protection, a cDNA segment corresponding to the 272 bp EVI-1 RT–PCR (Russell *et al.*, 1994) product was cloned into the PCII (Clontech, Palo Alto, CA, USA) vector and transcribed using Maxiscript (Ambion, Austin, TX, USA) *in vitro* transcription kit, according to the manufacturer's instructions. The resulting probe had an average specific activity of approximately $2\times10^8$ c.p.m. $\mu g^{-1}$ and was gel purified. The probe was hybridised with 10 $\mu$g total RNA from cell lines or 40 $\mu$g patient RNA using a RPAII (Ambion) ribonuclease (RNAase) protection assay kit. RNAase protection was performed using RNA from cell lines, HEL (positive control) and HL60 (negative control); ovarian carcinomas, SKOV-3, Colo-316, 367, 2780, 2008, UACC 1598, UACC 326, UACC 2727 and UACC 295; and prostate cancers, PC3 and DU-143. A previous study, using UCSD/AML-1 RNA showed RT–PCR to be approximately 300 times more sensitive than RNAase protection (Russell *et al.*, 1994). Using phosphor-imaging of the RNAase products (Molecular Dynamics, Sunnyvale, CA, USA), a linear relationship between signal intensity and the amount of RNA was present over at least a 1 log range of UCSD/AML1 RNA. A GAPDH control probe (Ambion) was labelled to approximately 50% of the activity of the EVI-1 probe, and hybridised in either a different or the same tube to cell line RNA. These results were used to verify RNA integrity and to normalise EVI-1 results in calculating RNA content.

RNA levels were also assessed by Northern analyses or slot blots as previously described (Oval *et al.*, 1992). Total cell RNA was extracted as described above and Northern or slot blots probed with a PCR-generated 272 bp probe, corresponding to the RNAase protection assay. Blots were normalised for RNA loading using a β-actin probe (Oval et al., 1992; Taetle et al., 1991). RNA half-lives were estimated by culturing cells with 10 μg $ml^{-1}$ actinomycin-D for 2 and 4 h as described (Oval et al., 1992). Responses to 10 μg/ml cycloheximide were assessed in a similar fashion.

*Southern blot analysis*

DNA prepared from normal donor lymphocytes, UCSD/AML1 cells (Oval et al., 1992), and carcinoma cell lines OVCAR3, SKOV-3, 2780, 2008, Colo-316, UACC 1598 and UACC 295 was digested with *Eco*RI, *Bam*HI or both enzymes and electrophoresed in 0.9% agarose gels. DNA products were transfered to nylon filters as previously described (Taetle et al., 1991) and hybridised with a PCR-generated, digoxinin-labelled probe to EVI-1 5′ flanking sequences from −486 to −974 base pairs (Madden et al., 1991); a 2.0 kb probe containing human 5′ flanking and coding sequences (Morishita et al., 1990b); and a full-length, murine EVI-1 cDNA (Oval et al., 1992). Blots were stripped and probed for actin to ensure DNA integrity. EVI-1 DNA content was expressed relative to normal ovary or lymphocyte DNA probed on the same blot.

*Karyotypes and fluoroscence* in situ *hybridisation (FISH)*

Cell line karyotypes were performed as described (Thompson et al., 1994). Whole chromosome 3 FISH was performed according to manufacturer's instructions (Oncor, Gaithersburg, MD, USA) (Pinkel et al., 1988). A human 3q26, band-specific probe was created by microdissection of normal chromosome 3 and labelled as previously described (Guan et al., 1995; Thompson et al., 1995). Hybridisation and FISH were performed as described previously (Guan et al., 1995; Thompson et al., 1995).

### Results

Previous studies using Northern anlayses suggested EVI-1 RNA expression was highly restricted among established human, non-haematological, tumour cell lines (Morishita et al., 1990b). In contrast, using RT−PCR, EVI-1 RNA products of 254 and 272 bp (because of alternate splicing) were detected in five of six established ovarian cancer cell lines. Cell line 2780 was consistently negative using RT−PCR direct at both 5′ (not shown) and 3′ EVI-1 coding sequences (Figure 1a), indicating truncated EVI-1 transcripts were not present. This result for 2780 cells was also verified using immunostaining and RNAase protection (see below). EVI-1 RNA expression was previously induced in primary kidney cells by cAMP (Bartholomew and Clark, 1994) and acute promyelocytic leukaemia cells by all-*trans* retinoic acid (Taetle et al., 1995). However, cAMP ($10^{-7}$ or $10^{-6}$ M) all-*trans* retinoic acid ($10^{-6}$ or $10^{-7}$ M) failed to induce EVI-1 RNA expression detected by RT−PCR in 2780 ovarian carcinoma cells. Similarly, 17 of 19 ovarian lines established within the Arizona Cancer Center and tested at passages <20 also expressed EVI-1 RNA (for example, Figure 1b). All cell lines positive for 3′ EVI-1 PCR products were also positive using 5′ product primers. The latter encompass the major start site and first 434 bp of the coding sequence. Thus all cells positive for EVI-1 RNA appeared to contain full-length transcripts.

Frozen primary tumour specimens (six ovarian epithelial and one ovarian sarcoma) were also positive for EVI-1 RNA using RT−PCR (Table I). Four of four ovaries obtained from premenopausal patients, which were histologically normal except for the presence of follicular cysts, contained low but detectable levels of EVI-1 RNA. RT−PCR of a normal post-menopausal (oocyte-poor) ovary did not contain EVI-1 RNA. RT−PCR was also used to assess EVI-1 expression in non-ovarian tumour cells and normal fibroblasts and was detected in the majority of these cells (Table I). Thus, in contrast to previous studies using Northern analysis (Morishita et al., 1990b), results using RT−PCR indicate EVI-1 RNA is widely expressed in human solid tumours and in normal ovary.

To verify the presence of the EVI-1 protein in ovarian tissues, immunostaining of frozen tissues and fresh cells was performed. Similar to studies of murine tissues (Perkins et al., 1991b), staining of two normal ovaries showed EVI-1 protein essentially limited to oocyte cytoplasm with only faint stromal staining (Figure 2a). In contrast, immunostaining of primary ovarian carcinomas was intense in cytoplasm and present in nuclei (Figure 2b), while controls (Figure 2c) did not stain. Cytospin preparations of an EVI-1-positive ovarian cell line, SKOV-3, showed discrete nuclear staining and

**Figure 1** (**a**) Detection of EVI-1 RNA by RT−PCR in established ovarian carcinoma cell lines: 3′ EVI-1 yields a doublet of 254 and 272 bp owing to alternate splicing. HL60 (lane 2) and HEL (lane 3) serve as negative and positive controls respectively. Ovarian cell lines from left to right in lanes 4−8 include SKOV3, 367, 2780, Colo-316 and 2008. Cell line 367 (lane 5) yields a barely detectable, but consistently positive, signal, and 2780 is consistently negative. Bottom panels show GAPDH controls. L, a 100 base pair ladder; lane 1, mock control. (**b**) RNA PCR for EVI-1 RNA in newly established cell cultures from ovarian carcinomas. HL60 and HEL again serve as controls (lanes 2 and 10 respectively). Lanes 3−8 are UACC cell line 1598, 326, 188, 2614, 1763 and 469. Bottom panel shows histone controls. Lane 1, mock reaction (no RNA).

**Table I** EVI-1 expression by RNA PCR

| | *EVI-1 RNA* |
|---|---|
| Cell lines | |
| Leukaemia/lymphoma (Russell et al., 1994) | 3/12 |
| Ovarian carcinoma | 22/25 |
| Melanoma | 6/7 |
| Endometrium (HEC1B) | 1/1 |
| Breast | 4/7 |
| Prostrate | 3/3 |
| Bladder | 1/1 |
| Epidermoid carcinoma | 1/1 |
| Primary tissues | |
| Normal ovary | 3/5 |
| Ovarian tumours[a] | 5/6 |
| Fibroblasts | 1/3[b] |
| Normal marrow (Russell et al., 1994) | 0/9 |

Presence of EVI-1 RNA by RT−PCR for tumour-derived cell lines, primary tumour and tissue specimens. Results from leukaemia and lymphoma specimens, fibroblasts and normal marrow have been previously reported (Russell et al., 1994). Normal ovarian tissue was obtained from patients who underwent surgery for non-metastatic endometrial cancer. [a]Five carcinomas and one-primary ovarian sarcoma. [b]Positive in fetal fibroblasts.

**Figure 2** (**a**) Oocyte cytoplasm from a normal ovary demonstrating intense staining for EVI-1 (original magnification ×400). (**b**) Metastatic ovarian adenocarcinoma (original magnification ×200). EVI-1 staining in the cytoplasm with focal nuclear positivity (counterstain, nuclear fast red). (**c**) Metastastic ovarian adenocarcinoma control slide (rabbit immunoglobulin), nuclear fast red counterstain. (**d**) SKOV-3 cell line (original magnification ×1000) shows punctate, primarily nuclear, staining for EVI-1 (no counterstain).

minimal or no cytoplasmic staining (Figure 2d). Staining of 2780 cells showed no EVI-1 protein (not shown). These results indicate that, in contrast to normal ovary, EVI-1 protein is increased and widely expressed in ovarian cancers showing both cytoplasmic and nuclear distribution.

To compare levels of EVI-1 RNA in normal ovaries and cancer cell lines, RNAase protection for EVI-1 RNA was performed (Russell *et al.*, 1994). EVI-1 RNA was not detected in 2780 or UACC 1598 cells, but was detected at equal levels using phosphor-imaging of RNA signals from normal ovaries and the 376 cell line. EVI-1 RNA was detected by RNAase protection in 11/13 ovarian cell lines and in HEL leukaemia cells (see Figure 3 for an example). EVI-1 RNA was also detected by RNAase protection in two out of three melanoma and two out of two prostate cell lines (not shown).

Using the phosphor-imaging and normalising to GAPDH levels, EVI-1 RNA expression by normal ovary was assigned a value of 1.0. Tumour RNA levels were determined on gels containing normal ovary or 367 RNA. Using this scale, HEL leukaemia cells expressed 2–4 times and UCSD/AML1 leukaemia cells seven times the level in normal ovary or 367 cells (not shown). Ovarian cell lines expressed 0 to 40 times the level found in the median of three normal ovaries (Table I). Expression by the SKOV-3 and UACC 295 cell lines exceeded linearity of the assay, but was estimated at 11–40 times that of normal ovary and at least four times that of HEL cells. Cell line UACC 1598 contained EVI-1 RNA detected by RNA PCR, but undetectable EVI-1 RNA by RNAase protection. PC3 prostate cancer cells contained EVI-1 RNA at levels apparently identical to SKOV-3 cells (not shown, two experiments). Using Northern blots, the half-life of EVI-1 RNA from SKOV-3 and Colo-316 cells in the

**Figure 3** Ribonuclease (RNAase) protection assay of EVI-1 RNA (left) or GADPH (right) in ovarian cancer lines. HL60, lane 1; HEL, lane 2. Left, SKOV-3 (lane 3) is overexpressed relative to all other lines. Ovarian cell lines 367, 2780, Colo-316, and 2008, lanes 4, 5, 6 and 7 respectively. 2780 (lane 5) is negative and Colo-316 (lane 4) is not visible by light photography but is consistently positive by phosphor-imaging. Digested and undigested yeast (lanes 8 and 9) are controls for RNAase assay integrity. EVI-1 expression is measured relative to GAPDH expression.

presence of actinomycin-D was 2.5 h, a value nearly identical to those previously obtained for EVI-1 transcripts in UCSD/AML1 leukaemia and HEC-1B endometrial carcinoma cells (Oval *et al.*, 1992). Levels of EVI-1 RNA 146–153% of control after exposure to 10 $\mu$g ml$^{-1}$ cycloheximide for 2 h (two studies).

Southern blots did not detect rearrangements or amplification of EVI-1 in ovarian carcinoma cell lines (Table II). In contrast, a rearrangement predicted from pulse-field maps (Oval *et al.*, 1992) was detected in UCSD/AML1 DNA digested with *Bam*HI/*Eco*RI. The intensity of EVI-1 bands detected on Southern analysis did not differ between normal diploid ovarian DNA, normal lymphocyte DNA and the ovarian tumours, indicating that the EVI-1 gene was not significantly amplified (Table I). Southern blots of 2780 and

Table II Cytogenetic evaluation and EVI-1 expression in ovarian cancer cell lines

| Cell line | Modal chromosome number | Karyotype | Chromosome 3 FISH | 3q26 FISH | EVI-1 DNA | EVI-1 RNA |
|---|---|---|---|---|---|---|
| OVCAR3 | 63–66 | Normal 3×2 qdp(3)(q24–q27) | Nml 3×2 qdp(3) | 7–12 3q26 signals/cell; translocation/ inversion of 3q26 | 1.3 | 10 |
| SKOV-3 | 71–74 | +del(3)(q24) | +der(3) | 4–6 translocations involving 3q26 | 1.2 | 40 |
| 367 | 49–56 | ND | ND | ND | 1.4 | 1 |
| 2008 | 58 | i(3)(p10) i(3)(q10) +del(3)(p14) | Same | No 3q26 translocations | ND | 6 |
| Colo-316 | 61–63 | i(3)(p10) del(3)(q21) 12 mars | add(3)(?) add(3)(q11) 9–10 markers contain chrom 3 sequences | Three translocations involving 3q26 | 1.2 | 8 |
| 2780 | 46 | t(X;3)(q24;p21) | Same | No 3q26 translocations | 1.0 | 0 |
| UACC 1598 | 57–66 dmin | Normal × 1 i(3q) × 1 der(8)t(3;8) × 1 der(3p)hsr(3p) | Same | One 3q26 translocation | 1.4 | 0 |
| UACC 295 | 61–67 | Normal 3×1 der(3)t(1;3) (q21;p21)×1 del(3pl3)add(3) (q31)×1 add(3)(p21)add(3) (q24)×1 | Same | 4–6 3q26 translocations | 1.2 | 11 |
| Normal cells | 46 | Normal 3×2 | Two normal chromosome 3 | Two normal signals | 1 | 1 |

Karyotypes and modes were determined as described previously (Thompson *et al.*, 1994). FISH was performed using a whole chromosome 3 paint or 3q26 band-specific probe. EVI-1 DNA was determined from Southern blots probed for multiple areas of the *EVI-1* gene. Results represent means of four experiments normalised to either normal ovary or lymphocyte DNA. EVI-1 RNA levels are expressed relative to normal ovary EVI-1 RNA content using RNAase protection (See Methods). ND, not done.

UACC 1598 carcinoma DNA probed with a full-length EVI-1 cDNA also showed no rearrangements or deletions, indicating that failure of these cells to express EVI-1 was not due to bi-allelic gene deletion.

In leukaemia cells, abnormal EVI-1 transcription is frequently accompanied by rearrangements involving chromosome 3q26, the site of the EVI-1 gene (Warrel *et al.*, 1983; Morishita *et al.*, 1992; Oval *et al.*, 1992). Karyotypes, whole chromosome 3 and 3q26 band-specific FISH were performed in nine ovarian cell lines (Table II). UCSD/AML-1 leukaemia cells known to contain t(3;3)(q21–26) were used as controls (Figure 4a). OVCAR-3 cells contained a [der(3)] [quadruplication(3)(q24–27)] in addition to two normal chromosome 3s. Whole chromosome 3 and 3q26 FISH confirmed that [der(3)] material was of chromosome 3 origin (Figure 4c). Other cell lines did not have karyotype abnormalities involving 3q26, but chromosome 3 and 3q26 FISH showed der(3) chromosomes in SKOV-3 (Figure 4b) and Colo-316 cells, which were not detected by karyotype (Table II). These findings suggest chromosome 3q26 rearrangements are involved in EVI-1 overexpression in these cells.

### Discussion

EVI-1 is a member of a large family of zinc finger, DNA-binding proteins (Schleif, 1988; O'Halloran, 1993). These genes are extremely common, and constitute up to 1% of some human chromosomes (O'Halloran, 1993). While some zinc finger genes are clearly transcription factors (e.g. retinoic acid receptors; Muscenski *et al.*, 1988) or function as tumour-suppressors (e.g. WT1; Madden *et al.*, 1991; Gessler *et al.*, 1990), the functions of many members of this gene family remain unknown.

Expression of EVI-1 was previously documented only in HEC1b endometrial carcinoma cells from which the human cDNA was cloned (Morishita *et al.*, 1990*b*). Using RT–PCR, EVI-1 RNA was not detected in normal blood or marrow mononuclear cells, but is expressed by acute myelogenous leukaemia blasts and cells from the marrow of myelodysplasia patients (Russell *et al.*, 1994; Morishita *et al.*, 1992; Oval *et al.*, 1992). Enforced EVI-1 expression in blood cells antagonises transcriptional regulation by the erythroid/ megakaryocytic-specific GATA-1 protein (Kreider *et al.*, 1993) and blocks erythropoietin-stimulated growth and *in vitro* red blood cell differentiation (Kreider *et al.*, 1993). A fusion gene containing full-length EVI-1 and generated by t(3;21) in AML also blocks haemopoietic cell line differentiation (Tanaka *et al.*, 1995). Recent studies suggest EVI-1 and EVI-1 fusion genes control c-*fos* transcription and that consensus EVI-1 binding sequences can act as transcriptional regulators (Tanaka *et al.*, 1994; Morishita *et al.*, 1995). These studies indicate EVI-1 controls or modifies expression of important genes in human cancers, and can control cell differentiation.

In contrast to previous studies using Northern analyses (Morishita *et al.*, 1990*b*), the present studies using RT–PCR and RNAase protection indicate EVI-1 RNA expression is common in human solid tumours. The RT–PCR assay was previously shown to be 300 times more sensitive than RNAase protection and was able to detect one positive luekaemia cell diluted in 1000 in normal marrow cells (Russell *et al.*, 1994). Thus, techniques used in the present studies are at least 3 logs more sensitive than those used in previous studies of human solid tumours (Morishita *et al.*, 1990*b*).

Immunostaining of primary ovarian tumours and cell lines indicates a relative redistribution of EVI-1 protein from cytoplasm of normal oocytes to increased nuclear and diffuse epithelial cytoplasmic locations in ovarian tumours. Thus, altered EVI-1 expression is accompanied by potential effects of protein redistribution and overexpression. RNAase

**Figure 4** Fluorescence *in situ* hybridisation (FISH) with a chromosome 3q26 probe generated using microdissection. (**a**) UCSD/AML1 leukaemia cells containing known t(3;3)(q21;q26). The probe detects previously demonstrated translocated 3q26 sequences containing EVI-1 (arrow) (Morishita *et al*., 1992; Oval *et al*., 1992). (**b**) SKOV-3 ovarian carcinoma cells with high EVI-1 RNA levels demonstrating multiple derivative chromosomes containing 3q26 sequences. Chromosomes shown by arrows were demonstrated to be abnormal der(3) by whole chromosome FISH and Giemsa prebanding. (**c**) OVCAR3 ovarian carcinoma cells demonstrating inversion/duplication of 3q26 sequences (arrows).

protection assays indicate high-level EVI-1 expression in ovarian carcinoma and other solid tumour cell lines compared with normal ovary, suggesting EVI-1 plays a role in ovarian tumour progression or pathogenesis. Southern analyses (Table II) indicate EVI-1 overexpression was not accompanied by EVI-1 gene amplification.

In leukaemia cells, EVI-1 transcription is activated by long-range translocations involving chromosome 3q26 segments 3′ or 5′ of the EVI-1 gene (Russell *et al*., 1993; Morishita *et al*., 1992). It is unclear over what distances chromosome 3 rearrangements can alter EVI-1 transcription, but one case of leukaemia with EVI-1 expression with a 3q26 rearrangement was mapped by pulse-field gels up to 900 kb 5′ and 3′ of the gene without detecting a rearrangement (Jagasia *et al*., 1994). Although further studies are required to delineate mechanisms controlling EVI-1 expression in ovarian carcinoma cells, the half-life of EVI-1 RNA was identical to values in acute leukaemia and endometrial carcinoma cells (Oval *et al*., 1992). Despite this finding, Southern analyses did not show EVI-1 amplification or rearrangements (Table II). These findings suggest EVI-1 expression is also regulated by transcription in ovarian cancer cells. Although only one ovarian carcinoma cell line with increased EVI-1 RNA showed a definite karyotype abnormality involving band 3q26, using FISH, lines with relative EVI-1 overexpression showed rearrangements using a 3q26 band-specific probe. These studies suggest that rearrangements involving 3q26 are involved in abnormal EVI-1 expression in non-haemopoietic tumours as well.

The role and regulation of EVI-1 in non-haematological tissues are unknown. Sequential expression of EVI-1 in ontogeny suggests a role in cell migration or adhesion (Perkins *et al*., 1991*b*). EVI-1 is also highly conserved among species and recent studies in nematodes indicate it is highly homologous to a zinc finger binding protein, egl-43, important in long-range migration of (hermaphrodite-specific) motor neurons (Garriga *et al*., 1993). Further studies investigating potential role(s) of EVI-1 by gene transfer in ovarian tumours are in progress.

**Acknowledgements**
This study was supported in part by NIH grants CA23074 and CA41183, and ACS grant DHP-126.

### References

BARTHOLOMEW C AND CLARK AM. (1994). Induction of two alternatively spliced evi-1 proto-oncogene transcripts by cAMP in kidney cells. *Oncogene*, **9**, 939–942.

BORING CC, SQUIRES TS, TONG T AND MJONTGOMERY S. (1994). Cancer statistics 1994. *CA. Cancer J. Clin*., **44**, 7–26.

BUICK RN, PULLANO R AND TRENT JM. (1985). Comparative properties of five human ovarian adenocarcinoma cell lines. *Cancer Res*., **45**, 3668–3676.

CHENG JQ, GODWIN AK, BELLACOSA A, TAGUCHI T, FRANKE TF, HAMILTON TC, TSICHLIS PN AND TESTA JR. (1992). *AKT2*, a putative oncogene encoding a member of a subfamily of protein-serine/threonine kinsaes, is amplified in human ovarian carcinomas. *Proc. Natl Acad. Sci. USA*, **89**, 9267–9271.

DELWEL R, FUNABIKI T, KREIDER BL, MORISHITA K AND IHLE JN. (1993) Four of the seven zinc fingers of the *Evi-1* myeloid-transforming gene are required for sequence-specific binding to GA(C/T)AAGA(T/C)AAGATAA. *Mol. Cell. Biol.*, **13**, 4291–4300.

GARRIGA G, GUENTHER C AND HORVITZ HR. (1993). Migrations of the *Caenorhabditis elegans* HSNs are regulated by egl-43, a gene encoding two zinc finger proteins. *Genes Devel.*, **7**, 2097–2109.

GESSLER M, POUSTKA A, CAVANEE W, NEVE RL, ORKIN SH AND BRUNS GA. (1990). Homozygous deletion in Wilms' tumours of a zinc-finger gene identified by chromosome jumping. *Nature*, **343**, 774–778.

GUAN X-Y, CARGILE CB, ANZICK SL, THOMPSON FH, MELTZER PS, BITTNER ML, TAETLE R, MCGILL JR AND TRENT JM. (1995). Chromosome microdissection identifies cryptic sites of DNA sequence amplification in human ovarian carcinoma. *Cancer Res.*, **55**, 3380–3385.

GUESDON JL, TERNYNCK T AND AVRAMEAS SJ. (1995). The use of avidin–biotin interaction in immunoenzymatic techniques. *J. Histochem. Cytochem.*, **27**, 1131–1138.

JAGASIA S, PARGANAS E, GAJJAR A AND IHLE JN. (1994). Characterization of the translocations and inversions associated with activation of the EV11 gene in AML. *Blood*, **84**, 905.

KREIDER BL, ORKIN SH AND IHLE JN. (1993). Loss of erythropoietin responsiveness in erythroid progenitors due to expression of the EVI-1 myeloid-transforming gene. *Proc. Natl Acad. Sci. USA*, **90**, 6454–6458.

LEIBOWITZ A. (1986). Development of tumour cell lines. *Cancer Genet. Cytogenet.*, **19**, 11–19.

MADDEN SL, COOK DM, MORRIS JF, GASHLER A, SUKHATME VP AND RAUSCHER FJ III. (1991). Transcriptional repression mediated by WT1 Wilms' tumour gene product. *Science*, **253**, 1550–1553.

MANN SC, ANDREWS PA AND HOWELL SB. (1991). Modulation of *cis*-diamminedichloroplatinum (II) accumulation and sensitivity by forskolin and 3-isobutyl-1-methylxanthine in sensitive and resistant human ovarian carcinoma cells. *Int. J. Cancer*, **48**, 866–872.

MARKS JR, DAVIDOFF AM, KERNS BJ, HUMPHREY PA, PENCE JC, DODĠE RK, CLARKE-PEARSON DL, IGLEHART JD, BAST JR RC AND BERCHUCK A. (1991). Overexpression and mutation of p53 in epithelial ovarian cancer. *Cancer Res.*, **51**, 2979–2984.

MATSUGI T, MORISHITA K AND IHLE JN. (1990). Identification, nuclear localization and DNA-binding activity of zinc finger protein encoded by the evi-1 myeloid transforming gene. *Mol. Cell. Biol.*, **10**, 1259–1264.

MICKEY DD, STONE KR, WUNDERLI H, MICKEY GH AND PAULSON DF. (1980). Characterization of a human prostate adenocarcinoma cell line. *Prog. Clin. Biol. Res.*, **37**, 67–84.

MILNER BJ, ALLAN LA, ECCLES DM, KITCHENER HC, LEONARD RCF, KELLY KF, PARKIN DE AND HAITES NE. (1993). *p53* mutation is a common genetic event in ovarian carcinoma. *Cancer Res.*, **53**, 2128–2132.

MORISHITA K, PARGANAS E, BARTHOLOMEW C, SACCHI N, VALENTINE MB, RAIMONDI SC, LEBEAU MM AND IHLE JN. (1990*a*). The human evi-1 gene is located on chromosome 3q24–28 but is not rearranged in three cases of acute nonlymphocytic leukemias containing t(3;5)(q25;q34) translocations. *Oncogene Res.*, **5**, 221–231.

MORISHITA K, PARGANAS E, DOUGLASS EC AND IHLE JN. (1990*b*). Unique expression of the human evi-1 gene in an endometrial carcinoma cell line: sequence of cDNAs and structure of alternatively spliced transcripts. *Oncogene*, **5**, 963–971.

MORISHITA K, PARGANAS E, PARHAM DM, MATSUGI T AND IHLE JN. (1990*c*). The *Evi-1* zinc finger myeloid transforming gene is normally expressed in the kidney and in developing oocytes. *Oncogene*, **5**, 1419–1423.

MORISHITA K, PARGANAS E, WILLMAN CL, WHITTAKER MH, DRABKIN H, OVAL J, TAETLE R AND IHLE JN. (1992). Activation of evi-1 gene expression in human acute myelogenous leukemias by rearrangements spanning 300–400 kb on chromosome 3q26. *Proc. Natl Acad. Sci. USA*, **89**, 3937–3941.

MORISHITA K, SUZUKAWA K, TAKI T, IHLE JN AND YOKOTA J. (1995). EVI-1 zinc finger protein works as a transcriptional activator via binding to a consensus sequence of GACAAGATAA-GATAAN$_{1-28}$CTCATCTTC. *Oncogene*, **10**, 1961–1967.

MUCENSKI ML, TAYLOR BA, IHLE JN, HARTLEY JW, MORSE HC III, JENKINS NA AND COPELAND NG. (1988). Identification of a common ecotropic viral integration site, evi-1, in the DNA of AKXD murine myeloid tumours. *Mol. Cell. Biol.*, **8**, 301–308.

NAREDI P, HEATH DD, ENNS RE AND HOWELL SB. (1994). Cross-resistance between cisplatin and antimony in a human ovarian carcinoma cell line. *Cancer Res.*, **54**, 6464–6468.

O'HALLORAN TV. (1993). Transition metals in control of gene expression. *Science*, **261**, 715–725.

OVAL J, JONES OW, MONTOYA M AND TAETLE R. (1990). Characterization of a factor-dependent acute leukemia cell line with translocation (3;3)(q21;q26). *Blood*, **76**, 1369–1374.

OVAL J, SMEDSRUD M AND TAETLE R. (1992). Expression and regulation of the evi-1 gene in the human factor-dependent leukemia cell lines, UCSD/AML1. *Leukemia*, **6**, 446–451.

PERKINS AS, FISHEL R, JENKINS NA AND COPELAND NG. (1991*a*). *Evi-1*, a murine zinc finger proto-oncogene, encodes a sequence-specific DNA-binding protein. *Mol. Cell. Biol.*, **11**, 2665–2674.

PERKINS AS, MERCER JA, JENKINS NA AND COPELAND NG. (1991*b*). Patterns of *Evi-1* expression in embyronic and adult tissues suggest that *Evi-1* plays an important regulatory role in mouse development. *Development*, **111**, 479–487.

PINKEL D, LANDEGENT J, COLLINS C, FUSCOE J, SEAGRAVES R, LUCAS J AND GRAY J. (1988). Fluorescence *in situ* hybridization with human chromosome-specific libraries: detection of trisomy 21 and translocations of chromosome 4. *Proc. Natl. Acad. Sci. USA*, **85**, 9138–9142.

RODAN SB, INSOGNA KL, VIGNERY AM, STEWART AF, BROADUS AE, D'SOUZA SM, BERTOLINI DR, MUNDY GR AND RODAN GA. (1983). Factors associated with humoral hypercalcemia of malignancy stimulate adenylate cyclase in osteoblastic cells. *J. Clin. Invest.*, **72**, 1511–1515.

RUBIN SC, FINSTAD CL, WONG GY, ALMADRONES L, PLANTE M AND LLOYD KO. (1993). Prognostic significance of HER-2/*neu* expression in advanced epithelial ovarian cancer: a multivariate analysis. *Am. J. Obstet. Gynecol.*, **168**, 162–169.

RUBIN SC, FINSTAD CL, FEDERICI MG, SCHEINER L, LLOYD KO AND HOSKINS WJ. (1994). Prevalence and significance of HER-2/*neu* expression in early epithelial ovarian cancer. *Cancer*, **73**, 1456–1466.

RUSSELL M, THOMPSON F, SPIER C AND TAETLE R. (1993). Expression of the EVI1 gene in chronic myelogenous leukemia in blast crisis. *Leukemia*, **7**, 1654–1657.

RUSSELL M, LIST A, GREENBERG P, WOODWARD S, GLINSMANN B, PARGANAS E, IHLE J AND TAETLE R. (1994). Expression of EVI1 in myelodysplastic syndromes and other hematologic malignancies without 3q26 translocations. *Blood*, **84**, 1243–1248.

SASANO H, GARRETT CT, WILKINSON DS, SILVERBERG S, COMERFORD J AND HYDE J. (1990). Protooncogene amplification and tumour ploidy in human ovarian neoplasms. *Hum. Pathol.*, **21**, 382–391.

SCHLEIF R. (1988). DNA binding by proteins. *Science*, **241**, 1182–1187.

SMITH DM, GROFF DE, POKUL RK, BEAR JL AND DELGADO G. (1989). Determination of cellular oncogene rearrangement or amplification in ovarian adenocarcinomas. *Am. J. Obstet. Gynecol.*, **161**, 911–915.

TAETLE R AND HONEYSETT JM. (1987). Effects of monoclonal anti-transferrin receptor antibodies on in vitro growth of human solid tumor cells. *Cancer Res.*, **47**, 2040–2044.

TAETLE R, JONES OW, HONEYSETT JM, ABRAMSON I, BRADSHAW C AND REID S. (1987). Use of nude mouse xenografts as pre-clinical screens: characterization of xenograft-derived melanoma cell lines. *Cancer*, **60**, 1836–1841.

TAETLE R, HONEYSETT JM AND HOUSTON LL. (1988). Effects of anti-epidermal growth factor (EGF) receptor antibodies and an anti-EGF receptor recombinant-ricin A chain immunoconjugate on growth of human cells. *J. Natl Cancer Inst.*, **80**, 1053–1059.

TAETLE R, OVAL J, SMEDSRUD M, DAVIS C AND GANSBACHER B. (1991). Analysis of granulocyte-macrophage colony-stimulating factor action in differentiating myeloid leukemia cells: treatment with DMSO may reveal a common pathway for growth factor gene regulation. *Exp. Hematol.*, **19**, 213–220.

TAETLE R, RUSSELL M, DOS SANTOS B, WOODWARD S, XI ZF AND WAGNER L. (1995). Expression the EVI-1 Zn finger gene during normal myeloid and all-trans retinoic acid-induced promyelocytic leukemia differentiation. *Blood*, **86**, 189a.

TANAKA T, NISHIDA J, MITANI K, OGAWA S, YAZAKI Y AND HIRAI H. (1994). Evi-1 raises AP-1 activity and stimulates c-*fos* promoter transactivation with dependence on the second zinc finger domain. *J. Biol. Chem.*, **269**, 24020-24026.

TANAKA T, MITANI K, KUROKAWA M, OGAWA S, TANAKA K, NISHIDA J, YAZAKI Y, SHIBATA Y AND HIRAI H. (1995). Dual functions of the AML1/Evi-1 chimeric protein in the mechanism of leukemogenesis in 5(3;21) leukemias. *Mol. Cell. Biol.*, **15**, 2383-2392.

THOMPSON FH, EMERSON J, ALBERTS D, LIU Y, GUAN X-Y, BURGESS A, FOX S, TAETLE R, WEINSTEIN R, MAKAR R, POWELL D AND TRENT J. (1994). Clonal chromosome abnormalities in 54 cases of ovarian carcinoma. *Cancer Genet. Cytogenet.*, **73**, 33-45.

THOMPSON FH, NELSON MA, TRENT JM, GUAN X-Y, LIU Y, YANG J-M, EMERSON J, ADAIR L, WYMER J, BALFOUR C, MASSEY K, WEINSTEIN R, ALBERTS DS AND TAETLE R. (1995). Amplification of 19q13.1-q13.2 sequences in ovarian cancer: G-band, FISH, and molecular studies. *Cancer Genet. Cytogenet.*, **87**, 55-62.

VAN'T VEER LJ, HERMENS R, VAN DEN BERG-BAKKAR LAM, CHENG NC, FLEUREN G-J, BOS JL, CLETON FJ AND SCHRIER PI. (1988). *ras* oncogene activation in human ovarian carcinoma. *Oncogene*, **2**, 157-165.

WARREL RP JR, COONLEY CJ, STRAUS DJ AND YOUNG CW. (1983). Treatment of patients with advanced malignant lymphoma using gallium nitrate administered as a seven-day continuous infusion. *Cancer*, **51**, 1982-1987.

WILDING G, GELMANN EP AND FRETER CE. (1990). Phosphoinositide metabolism in human prostate cancer cells *in vitro*. *Prostate*, **16**, 15-27.

WOODS LK, MORGAN RT, QUINN LA, MOORE GE, SEMPLE TU AND STEDMAN KE. (1979). Comparison of four new cell lines from patients with adenocarcinoma of the ovary. *Cancer Res.*, **39**, 4449-4459.


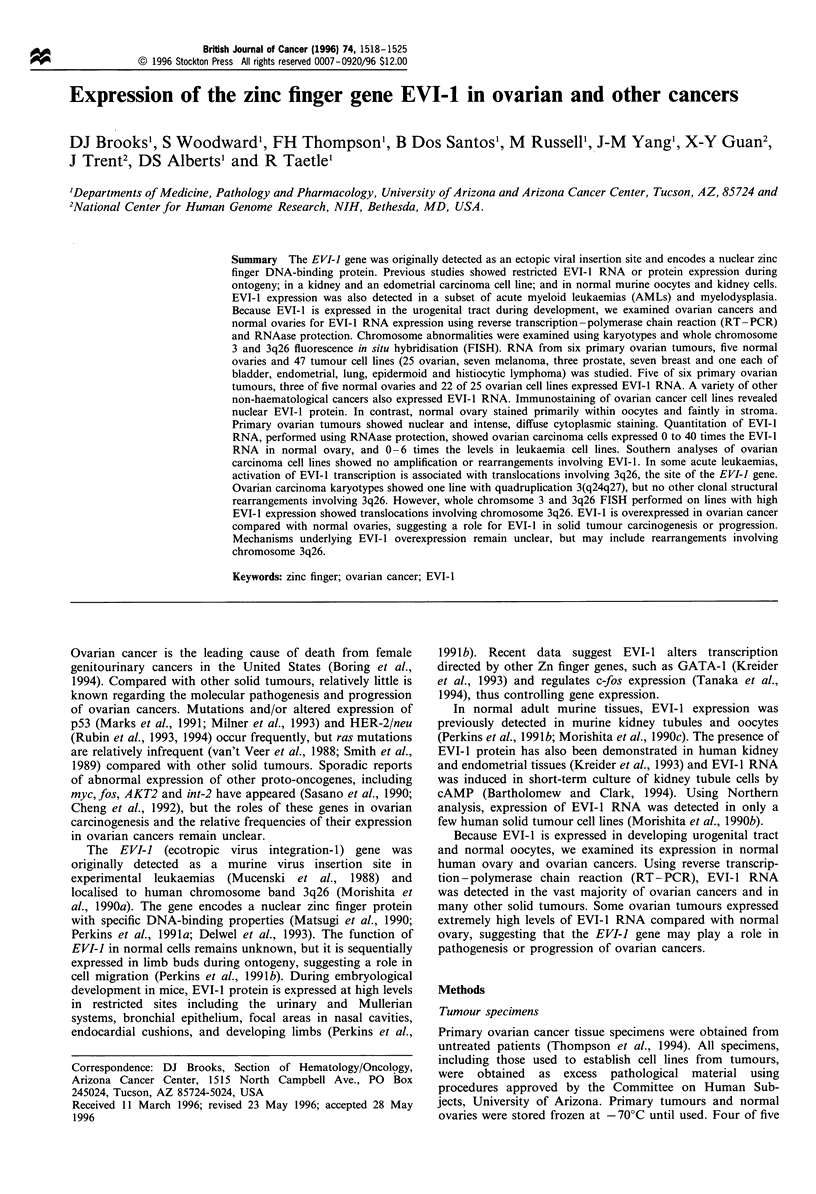

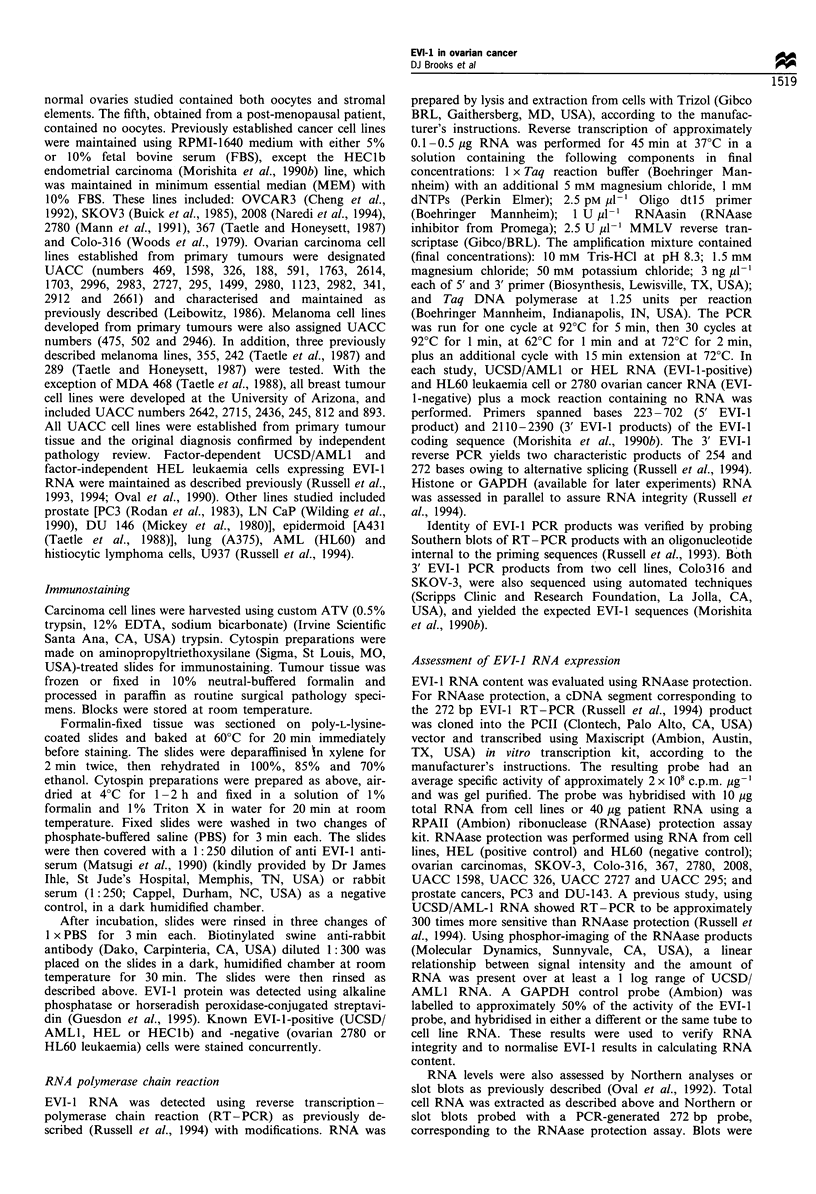

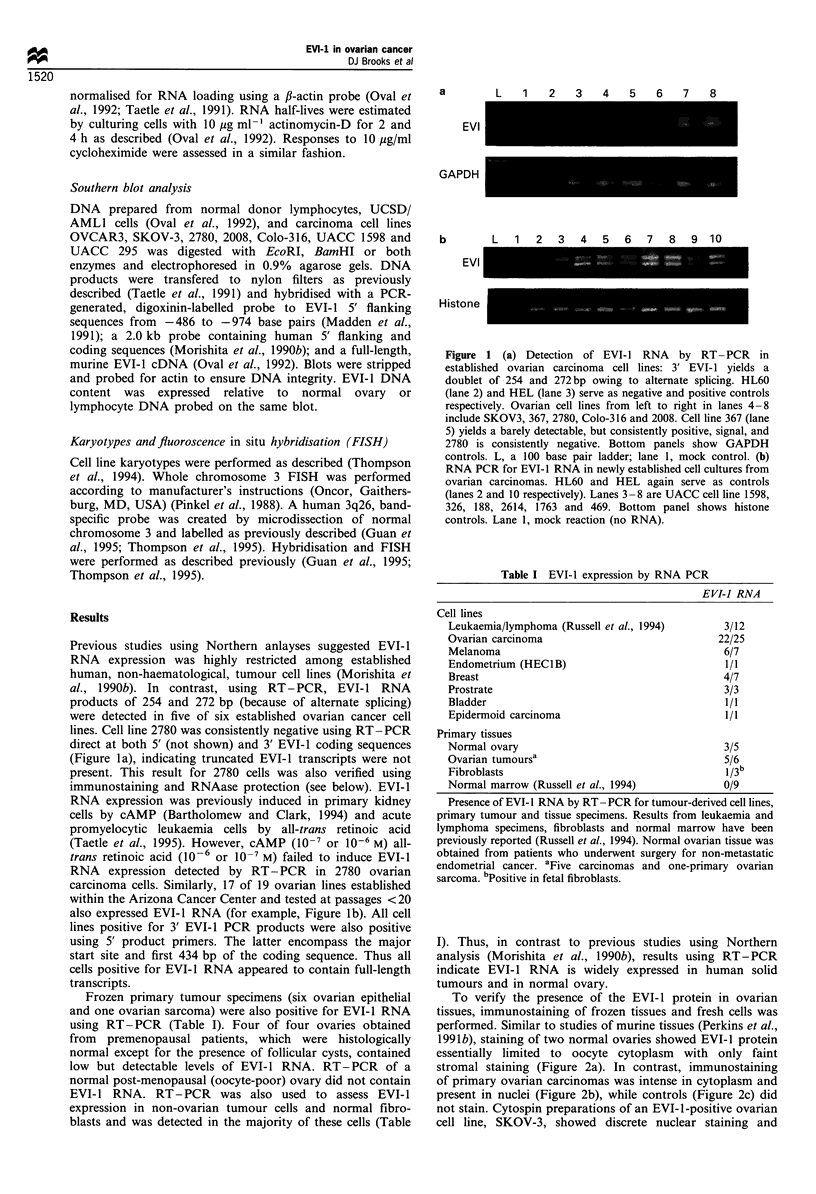

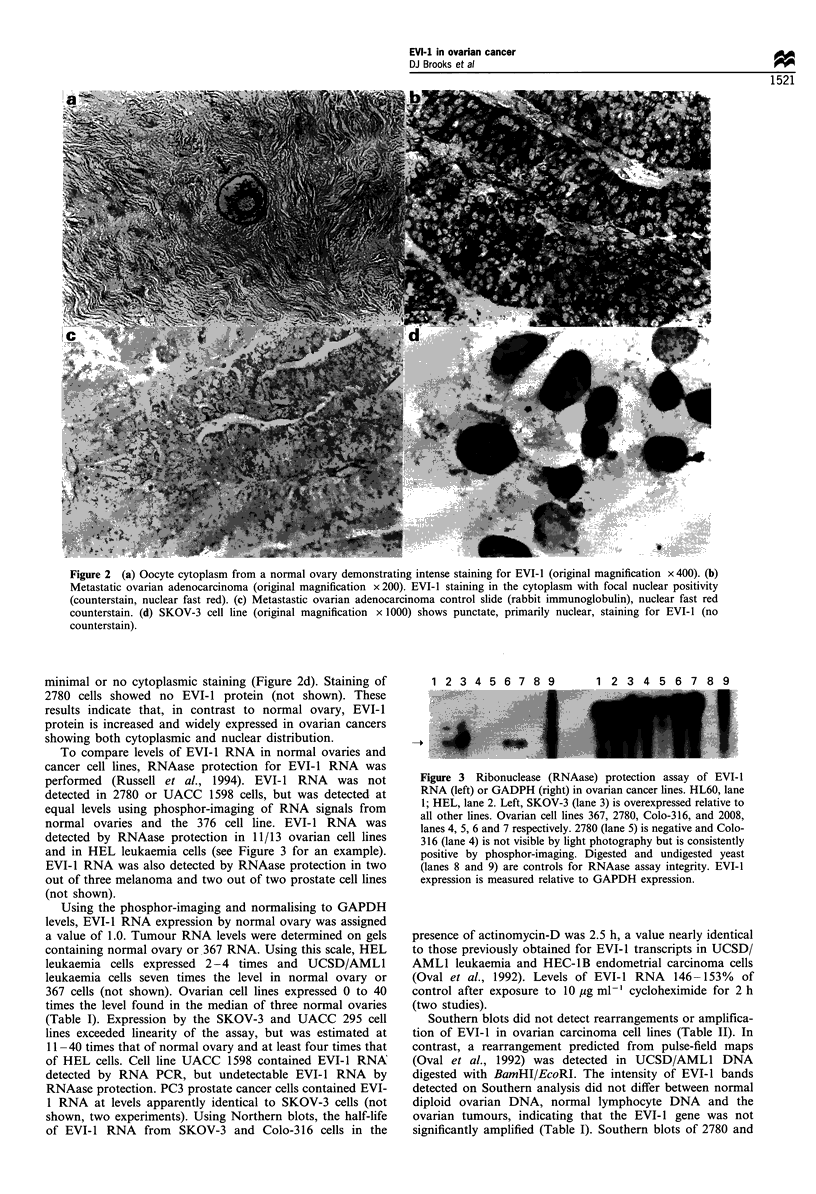

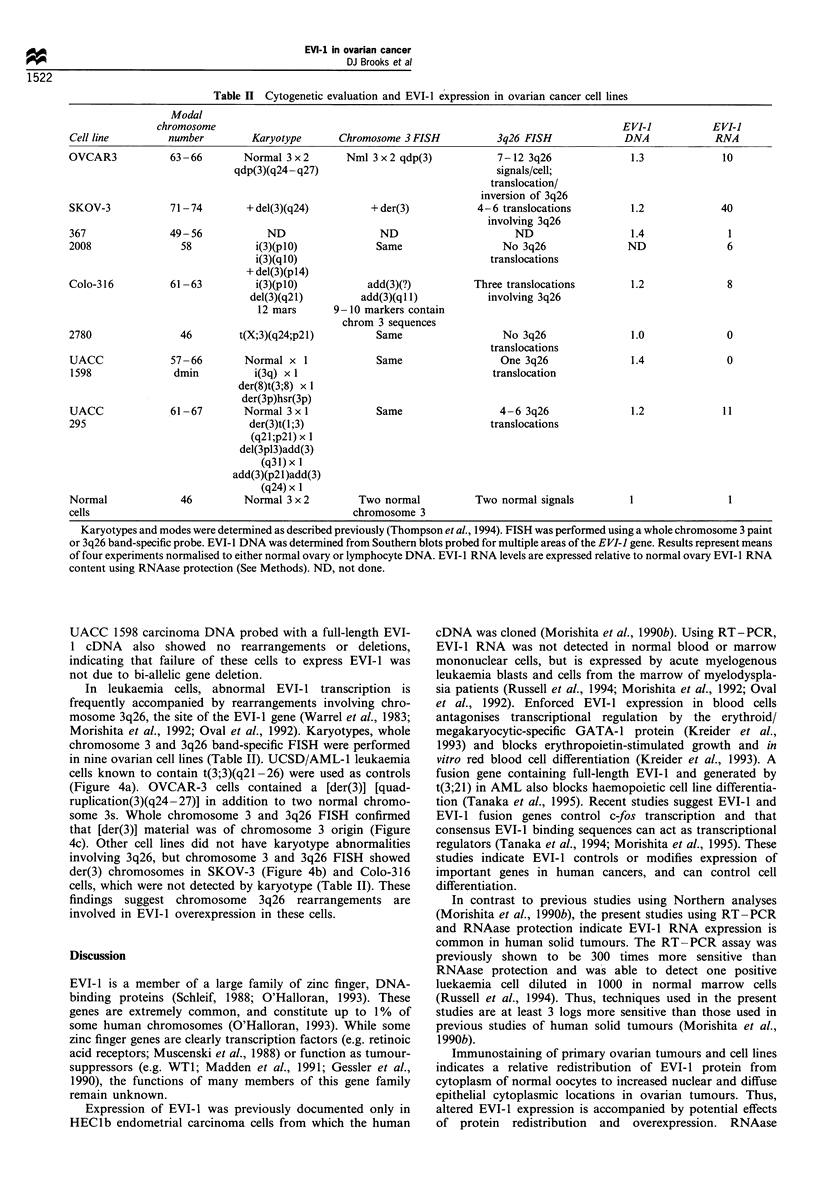

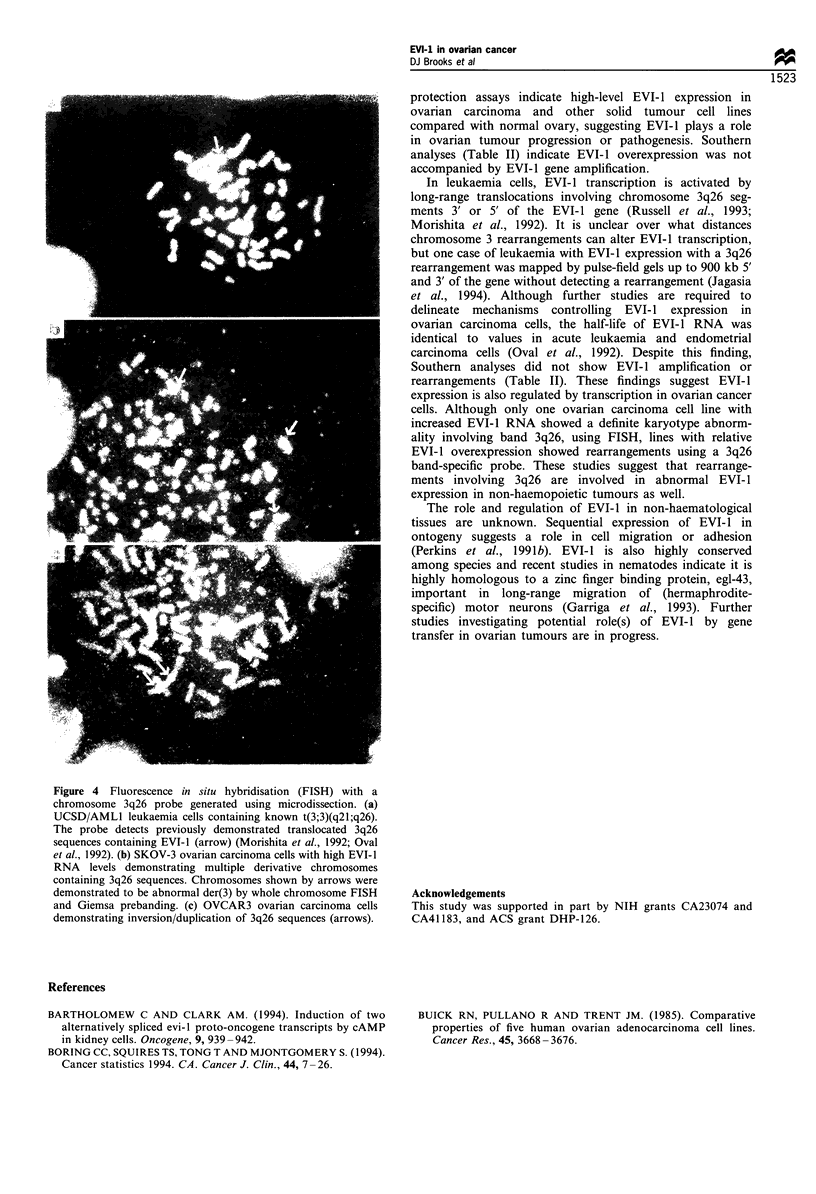

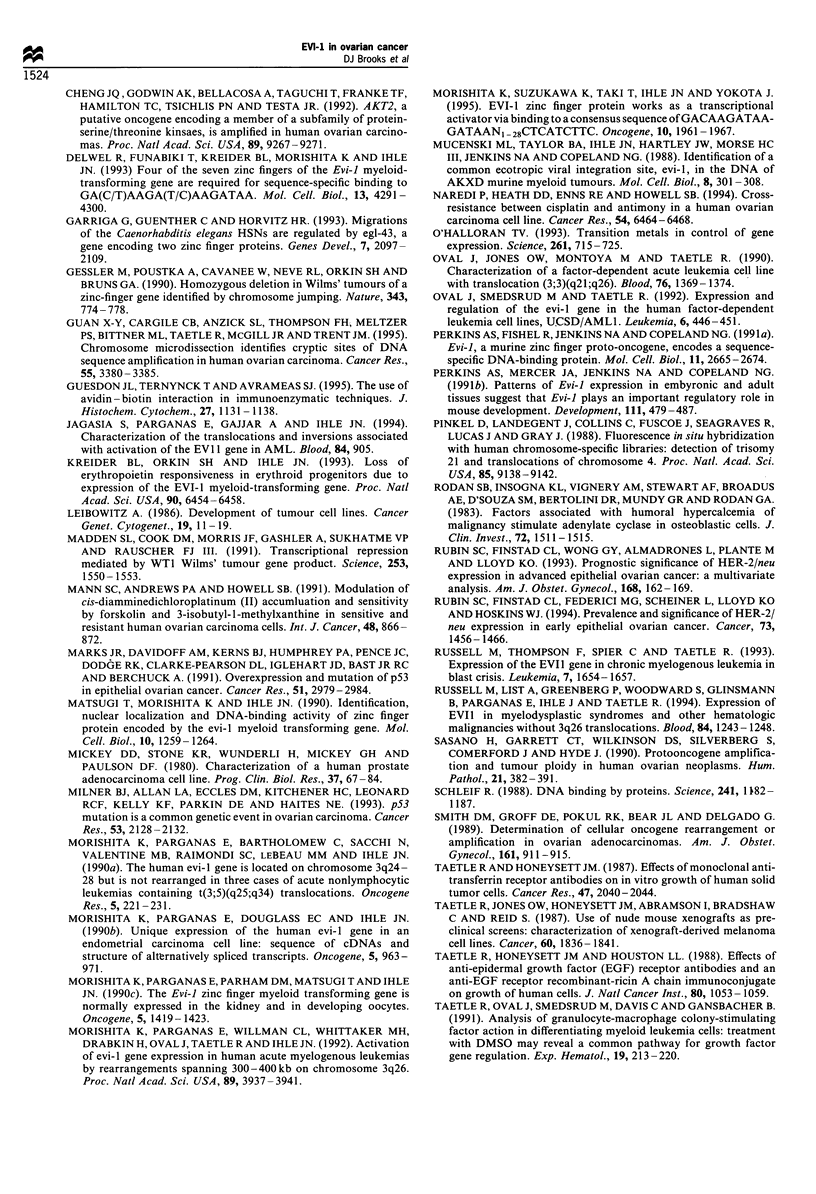

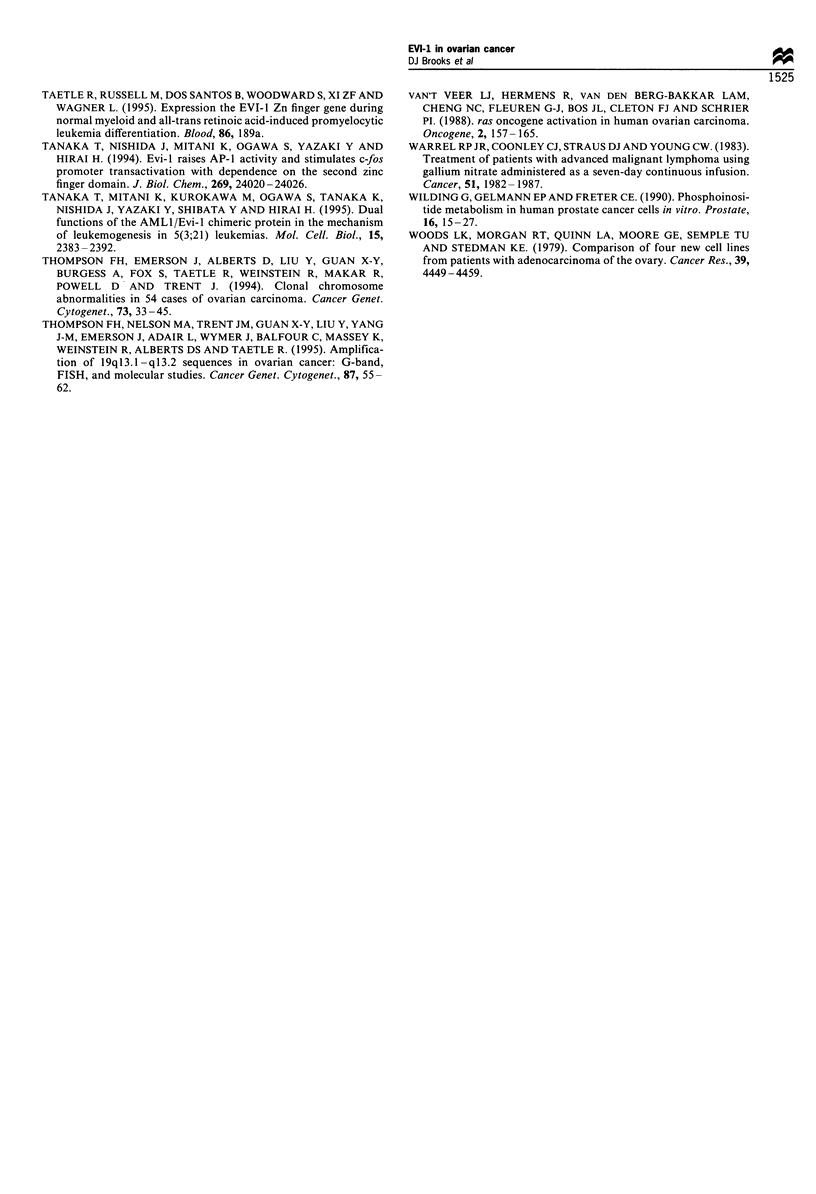


## References

[OCR_00720] Bartholomew C., Clark A. M. (1994). Induction of two alternatively spliced evi-1 proto-oncogene transcripts by cAMP in kidney cells.. Oncogene.

[OCR_00725] Boring C. C., Squires T. S., Tong T., Montgomery S. (1994). Cancer statistics, 1994.. CA Cancer J Clin.

[OCR_00732] Buick R. N., Pullano R., Trent J. M. (1985). Comparative properties of five human ovarian adenocarcinoma cell lines.. Cancer Res.

[OCR_00739] Cheng J. Q., Godwin A. K., Bellacosa A., Taguchi T., Franke T. F., Hamilton T. C., Tsichlis P. N., Testa J. R. (1992). AKT2, a putative oncogene encoding a member of a subfamily of protein-serine/threonine kinases, is amplified in human ovarian carcinomas.. Proc Natl Acad Sci U S A.

[OCR_00749] Delwel R., Funabiki T., Kreider B. L., Morishita K., Ihle J. N. (1993). Four of the seven zinc fingers of the Evi-1 myeloid-transforming gene are required for sequence-specific binding to GA(C/T)AAGA(T/C)AAGATAA.. Mol Cell Biol.

[OCR_00755] Garriga G., Guenther C., Horvitz H. R. (1993). Migrations of the Caenorhabditis elegans HSNs are regulated by egl-43, a gene encoding two zinc finger proteins.. Genes Dev.

[OCR_00761] Gessler M., Poustka A., Cavenee W., Neve R. L., Orkin S. H., Bruns G. A. (1990). Homozygous deletion in Wilms tumours of a zinc-finger gene identified by chromosome jumping.. Nature.

[OCR_00768] Guan X. Y., Cargile C. B., Anzick S. L., Thompson F. H., Meltzer P. S., Bittner M. L., Taetle R., McGill J. R., Trent J. M. (1995). Chromosome microdissection identifies cryptic sites of DNA sequence amplification in human ovarian carcinoma.. Cancer Res.

[OCR_00772] Guesdon J. L., Ternynck T., Avrameas S. (1979). The use of avidin-biotin interaction in immunoenzymatic techniques.. J Histochem Cytochem.

[OCR_00782] Kreider B. L., Orkin S. H., Ihle J. N. (1993). Loss of erythropoietin responsiveness in erythroid progenitors due to expression of the Evi-1 myeloid-transforming gene.. Proc Natl Acad Sci U S A.

[OCR_00795] Madden S. L., Cook D. M., Morris J. F., Gashler A., Sukhatme V. P., Rauscher F. J. (1991). Transcriptional repression mediated by the WT1 Wilms tumor gene product.. Science.

[OCR_00800] Mann S. C., Andrews P. A., Howell S. B. (1991). Modulation of cis-diamminedichloroplatinum(II) accumulation and sensitivity by forskolin and 3-isobutyl-1-methylxanthine in sensitive and resistant human ovarian carcinoma cells.. Int J Cancer.

[OCR_00808] Marks J. R., Davidoff A. M., Kerns B. J., Humphrey P. A., Pence J. C., Dodge R. K., Clarke-Pearson D. L., Iglehart J. D., Bast R. C., Berchuck A. (1991). Overexpression and mutation of p53 in epithelial ovarian cancer.. Cancer Res.

[OCR_00813] Matsugi T., Morishita K., Ihle J. N. (1990). Identification, nuclear localization, and DNA-binding activity of the zinc finger protein encoded by the Evi-1 myeloid transforming gene.. Mol Cell Biol.

[OCR_00820] Mickey D. D., Stone K. R., Wunderli H., Mickey G. H., Paulson D. F. (1980). Characterization of a human prostate adenocarcinoma cell line (DU 145) as a monolayer culture and as a solid tumor in athymic mice.. Prog Clin Biol Res.

[OCR_00822] Milner B. J., Allan L. A., Eccles D. M., Kitchener H. C., Leonard R. C., Kelly K. F., Parkin D. E., Haites N. E. (1993). p53 mutation is a common genetic event in ovarian carcinoma.. Cancer Res.

[OCR_00831] Morishita K., Parganas E., Bartholomew C., Sacchi N., Valentine M. B., Raimondi S. C., Le Beau M. M., Ihle J. N. (1990). The human Evi-1 gene is located on chromosome 3q24-q28 but is not rearranged in three cases of acute nonlymphocytic leukemias containing t(3;5)(q25;q34) translocations.. Oncogene Res.

[OCR_00838] Morishita K., Parganas E., Douglass E. C., Ihle J. N. (1990). Unique expression of the human Evi-1 gene in an endometrial carcinoma cell line: sequence of cDNAs and structure of alternatively spliced transcripts.. Oncogene.

[OCR_00846] Morishita K., Parganas E., Parham D. M., Matsugi T., Ihle J. N. (1990). The Evi-1 zinc finger myeloid transforming gene is normally expressed in the kidney and in developing oocytes.. Oncogene.

[OCR_00852] Morishita K., Parganas E., William C. L., Whittaker M. H., Drabkin H., Oval J., Taetle R., Valentine M. B., Ihle J. N. (1992). Activation of EVI1 gene expression in human acute myelogenous leukemias by translocations spanning 300-400 kilobases on chromosome band 3q26.. Proc Natl Acad Sci U S A.

[OCR_00858] Morishita K., Suzukawa K., Taki T., Ihle J. N., Yokota J. (1995). EVI-1 zinc finger protein works as a transcriptional activator via binding to a consensus sequence of GACAAGATAAGATAAN1-28 CTCATCTTC.. Oncogene.

[OCR_00865] Mucenski M. L., Taylor B. A., Ihle J. N., Hartley J. W., Morse H. C., Jenkins N. A., Copeland N. G. (1988). Identification of a common ecotropic viral integration site, Evi-1, in the DNA of AKXD murine myeloid tumors.. Mol Cell Biol.

[OCR_00868] Naredi P., Heath D. D., Enns R. E., Howell S. B. (1994). Cross-resistance between cisplatin and antimony in a human ovarian carcinoma cell line.. Cancer Res.

[OCR_00873] O'Halloran T. V. (1993). Transition metals in control of gene expression.. Science.

[OCR_00879] Oval J., Jones O. W., Montoya M., Taetle R. (1990). Characterization of a factor-dependent acute leukemia cell line with translocation (3;3)(q21;q26).. Blood.

[OCR_00882] Oval J., Smedsrud M., Taetle R. (1992). Expression and regulation of the evi-1 gene in the human factor-dependent leukemia cell line, UCSD/AML1.. Leukemia.

[OCR_00889] Perkins A. S., Fishel R., Jenkins N. A., Copeland N. G. (1991). Evi-1, a murine zinc finger proto-oncogene, encodes a sequence-specific DNA-binding protein.. Mol Cell Biol.

[OCR_00894] Perkins A. S., Mercer J. A., Jenkins N. A., Copeland N. G. (1991). Patterns of Evi-1 expression in embryonic and adult tissues suggest that Evi-1 plays an important regulatory role in mouse development.. Development.

[OCR_00898] Pinkel D., Landegent J., Collins C., Fuscoe J., Segraves R., Lucas J., Gray J. (1988). Fluorescence in situ hybridization with human chromosome-specific libraries: detection of trisomy 21 and translocations of chromosome 4.. Proc Natl Acad Sci U S A.

[OCR_00908] Rodan S. B., Insogna K. L., Vignery A. M., Stewart A. F., Broadus A. E., D'Souza S. M., Bertolini D. R., Mundy G. R., Rodan G. A. (1983). Factors associated with humoral hypercalcemia of malignancy stimulate adenylate cyclase in osteoblastic cells.. J Clin Invest.

[OCR_00920] Rubin S. C., Finstad C. L., Federici M. G., Scheiner L., Lloyd K. O., Hoskins W. J. (1994). Prevalence and significance of HER-2/neu expression in early epithelial ovarian cancer.. Cancer.

[OCR_00915] Rubin S. C., Finstad C. L., Wong G. Y., Almadrones L., Plante M., Lloyd K. O. (1993). Prognostic significance of HER-2/neu expression in advanced epithelial ovarian cancer: a multivariate analysis.. Am J Obstet Gynecol.

[OCR_00932] Russell M., List A., Greenberg P., Woodward S., Glinsmann B., Parganas E., Ihle J., Taetle R. (1994). Expression of EVI1 in myelodysplastic syndromes and other hematologic malignancies without 3q26 translocations.. Blood.

[OCR_00924] Russell M., Thompson F., Spier C., Taetle R. (1993). Expression of the EVI1 gene in chronic myelogenous leukemia in blast crisis.. Leukemia.

[OCR_00934] Sasano H., Garrett C. T., Wilkinson D. S., Silverberg S., Comerford J., Hyde J. (1990). Protooncogene amplification and tumor ploidy in human ovarian neoplasms.. Hum Pathol.

[OCR_00942] Schleif R. (1988). DNA binding by proteins.. Science.

[OCR_00946] Smith D. M., Groff D. E., Pokul R. K., Bear J. L., Delgado G. (1989). Determination of cellular oncogene rearrangement or amplification in ovarian adenocarcinomas.. Am J Obstet Gynecol.

[OCR_00952] Taetle R., Honeysett J. M. (1987). Effects of monoclonal anti-transferrin receptor antibodies on in vitro growth of human solid tumor cells.. Cancer Res.

[OCR_00963] Taetle R., Honeysett J. M., Houston L. L. (1988). Effects of anti-epidermal growth factor (EGF) receptor antibodies and an anti-EGF receptor recombinant-ricin A chain immunoconjugate on growth of human cells.. J Natl Cancer Inst.

[OCR_00958] Taetle R., Jones O. W., Honeysett J. M., Abramson I., Bradshaw C., Reid S. (1987). Use of nude mouse xenografts as preclinical screens. Characterization of xenograft-derived melanoma cell lines.. Cancer.

[OCR_00969] Taetle R., Oval J., Smedsrud M., Davis C., Gansbacher B. (1991). Analysis of granulocyte-macrophage colony-stimulating factor action in differentiating myeloid leukemia cells: treatment with DMSO may reveal a common pathway for growth factor gene regulation.. Exp Hematol.

[OCR_00992] Tanaka T., Mitani K., Kurokawa M., Ogawa S., Tanaka K., Nishida J., Yazaki Y., Shibata Y., Hirai H. (1995). Dual functions of the AML1/Evi-1 chimeric protein in the mechanism of leukemogenesis in t(3;21) leukemias.. Mol Cell Biol.

[OCR_00989] Tanaka T., Nishida J., Mitani K., Ogawa S., Yazaki Y., Hirai H. (1994). Evi-1 raises AP-1 activity and stimulates c-fos promoter transactivation with dependence on the second zinc finger domain.. J Biol Chem.

[OCR_01002] Thompson F. H., Emerson J., Alberts D., Liu Y., Guan X. Y., Burgess A., Fox S., Taetle R., Weinstein R., Makar R. (1994). Clonal chromosome abnormalities in 54 cases of ovarian carcinoma.. Cancer Genet Cytogenet.

[OCR_01006] Thompson F. H., Nelson M. A., Trent J. M., Guan X. Y., Liu Y., Yang J. M., Emerson J., Adair L., Wymer J., Balfour C. (1996). Amplification of 19q13.1-q13.2 sequences in ovarian cancer. G-band, FISH, and molecular studies.. Cancer Genet Cytogenet.

[OCR_01022] Warrell R. P., Coonley C. J., Straus D. J., Young C. W. (1983). Treatment of patients with advanced malignant lymphoma using gallium nitrate administered as a seven-day continuous infusion.. Cancer.

[OCR_01026] Wilding G., Gelmann E. P., Freter C. E. (1990). Phosphoinositide metabolism in human prostate cancer cells in vitro.. Prostate.

[OCR_01031] Woods L. K., Morgan R. T., Quinn L. A., Moore G. E., Semple T. U., Stedman K. E. (1979). Comparison of four new cell lines from patients with adenocarcinoma of the ovary.. Cancer Res.

[OCR_01017] van 't Veer L. J., Hermens R., van den Berg-Bakker L. A., Cheng N. C., Fleuren G. J., Bos J. L., Cleton F. J., Schrier P. I. (1988). ras oncogene activation in human ovarian carcinoma.. Oncogene.

